# Impact of the COVID-19 Pandemic on Loneliness and Social Isolation: A Multi-Country Study

**DOI:** 10.3390/ijerph18199982

**Published:** 2021-09-23

**Authors:** Roger O’Sullivan, Annette Burns, Gerard Leavey, Iracema Leroi, Vanessa Burholt, James Lubben, Julianne Holt-Lunstad, Christina Victor, Brian Lawlor, Mireya Vilar-Compte, Carla M. Perissinotto, Mark A. Tully, Mary Pat Sullivan, Michael Rosato, Joanna McHugh Power, Elisa Tiilikainen, Thomas R. Prohaska

**Affiliations:** 1 Institute of Public Health, D08 NH90 Dublin, Ireland; annette.burns@publichealth.ie; 2 The Bamford Centre, Ulster University, Coleraine BT52 1SA, UK; g.leavey@ulster.ac.uk (G.L.); mg.rosato@ulster.ac.uk (M.R.); 3 The Global Brain Health Institute, Trinity College Dublin, D02 PN40 Dublin, Ireland; Iracema.Leroi@tcd.ie (I.L.); lawlorba@tcd.ie (B.L.); 4 School of Nursing/School of Population Health, Faculty of Medical and Health Sciences, University of Auckland, Auckland 1010, New Zealand; vanessa.burholt@auckland.ac.nz; 5 Centre for Innovative Ageing, School of Health and Social Care, Faculty of Medicine, Health and Life Science, Swansea University, Singleton Park, Swansea SA2 8PP, UK; 6 School of Social Work, Boston College, Chestnut Hill, MA 02467, USA; lubben@bc.edu; 7 Department of Psychology, Brigham Young University, Provo, UT 84602, USA; julianne_holt-lunstad@byu.edu; 8 College of Health, Medicine and Life Sciences, Brunel University London, Uxbridge UB8 3PH, UK; christina.victor@brunel.ac.uk; 9 Research Center for Equitable Development (EQUIDE), Universidad Iberoamericana, Mexico City 01219, Mexico; mireya.vilar@ibero.mx; 10 Division of Geriatrics, University of California San Francisco, San Francisco, CA 94143, USA; carla.perissinotto@ucsf.edu; 11 Institute of Mental Health Sciences, School of Health Sciences, Ulster University, Newtownabbey BT37 0QB, UK; m.tully@ulster.ac.uk; 12 School of Social Work, Faculty of Education and Professional Studies, Nipissing University, North Bay, ON P1B 8L7, Canada; maryps@nipissingu.ca; 13 Department of Psychology, Maynooth University, W23 F2K8 Kildare, Ireland; Joanna.MchughPower@mu.ie; 14 Department of Social Sciences, University of Eastern Finland, FI-70211 Kuopio, Finland; elisa.tiilikainen@uef.fi; 15 College of Health and Human Services, George Mason University, Fairfax, VA 22030, USA; tprohask@gmu.edu

**Keywords:** loneliness, social isolation, public health, COVID-19, risk factors

## Abstract

The COVID-19 global pandemic and subsequent public health social measures have challenged our social and economic life, with increasing concerns around potentially rising levels of social isolation and loneliness. This paper is based on cross-sectional online survey data (available in 10 languages, from 2 June to 16 November 2020) with 20,398 respondents from 101 different countries. It aims to help increase our understanding of the global risk factors that are associated with social isolation and loneliness, irrespective of culture or country, to support evidence-based policy, services and public health interventions. We found the prevalence of severe loneliness was 21% during COVID-19 with 6% retrospectively reporting severe loneliness prior to the pandemic. A fifth were defined as isolated based on their usual connections, with 13% reporting a substantial increase in isolation during COVID-19. Personal finances and mental health were overarching and consistently cross-cutting predictors of loneliness and social isolation, both before and during the pandemic. With the likelihood of future waves of COVID-19 and related restrictions, it must be a public health priority to address the root causes of loneliness and social isolation and, in particular, address the needs of specific groups such as carers or those living alone.

## 1. Introduction

The COVID-19 (SARS-CoV-2) global pandemic and subsequent public health measures which limited in-person social contact have impacted social and economic life with increasing concerns about physical and mental health [1,2,3]. Personal connections have been severely restricted or stopped completely, as have many connections with community and national services. Many people have disconnected from friends and family for long time periods in addition to a discontinuation of social and community engagements. Human beings are inherently social and cooperative, a predisposition that has assisted survival and development [4]. Therefore, as a result of the pandemic, we should expect major impacts on individual and community wellbeing. However, even prior to the pandemic, there was growing concern about an “epidemic of loneliness”, especially among Western and advanced economies [4].

Loneliness is commonly defined as an unpleasant experience or state of varying duration for an individual that arises when their relationship or social network is perceived as lacking in some way [5]. Social isolation is the objective experience of few meaningful social relationships and social contact with others [6]. Various explanations have been posited for loneliness and social isolation, varyingly situated within complex and intersecting social, cultural, psychological and economic factors [4,7].

While loneliness, rather than social isolation, has consumed much more recent interest, the separate (although sometimes overlapping) concepts both negatively impact health [8]. Recognizing that there are three main types of loneliness—social, emotional and existential—this paper focuses on the general concept to illustrate risk and protective factors [6,9,10]. The involuntary dimension and consideration of existential suffering are intriguing aspects of loneliness that occupy much of the related theory [11,12] and were of interest to this study. Would the pandemic and lockdown heighten the loneliness of those already at risk or make no difference? Do threats to social networks offered by employment tip people into loneliness or does pre-existing social capital in the form of community and religion provide protection? Are people more resilient than was anticipated and why [13]?

In response to the public health restrictions, a number of studies have examined the impact on loneliness and/or social isolation and the importance of specific risk factors including previous mental health problems, younger age, and being female [14,15,16,17,18,19,20,21,22,23]. However, much of this evidence is limited by measurement issues such as relying on single scales or items [22] and measuring aspects of loneliness but not social networks [16,17]. Additionally, most are restricted to single-country surveys [16,17,22], and/or including relatively small sample sizes and do not always collect pre COVID-19 data [18,19,23,24,25,26]. There is a need for transnational studies that use validated measures of loneliness and social isolation, collecting data for these outcomes pre-COVID-19, including the breadth of contextual factors that may attenuate or exacerbate loneliness and/or social isolation: socio-demographic, health and health behaviours, community, access and satisfaction with digital communication and COVID-19-specific experiences of bereavement and personal experiences of COVID-19.

To address these evidence gaps as well as support evidence-based policy, services and public health interventions, our overall aims were to (1) examine factors associated with increased loneliness and social isolation during the COVID-19 pandemic and (2) identify which global-level risk factors are most strongly associated with these outcomes, irrespective of culture or country. We sought to: (a) detect any increase in loneliness and social isolation during 2020 compared to pre-pandemic levels; (b) understand health and social impacts of loneliness and social isolation during the pandemic; (c) identify the core and consistent global risk factors for loneliness and social isolation both before and during the pandemic. We hypothesized that (a) the prevalence of loneliness in 2020 will be higher than prior to the pandemic; (b) socioeconomic factors will be associated with loneliness but not social isolation; (c) informal carers will be at increased risk of loneliness and social isolation during the COVID-19 pandemic compared to non-caregivers.

## 2. Methods

This paper is based on a cross-sectional, online multi-national study ‘Coping with Loneliness and Isolation during COVID-19’ (CLIC) (https://publichealth.ie/clic/ accessed on 30 July 2021). The survey methodology and content were derived through consensus by the International Loneliness and Isolation research Network (I-LINK) (https://publichealth.ie/ilink/ accessed on 30 July 2021), who are international academic experts in loneliness, isolation, and ageing. The risk factors and other items included in the survey were identified through workshops with international experts on loneliness and through the relevant literature. The survey consisted of 129 questions over five sections covering (1) socioeconomic and demographic factors; (2) health and health behaviours; (3) experiences of loneliness and social isolation before and during the COVID-19 pandemic; (4) social connections with family and friends; and (5) personal experiences of the COVID-19 pandemic. Prior to launch, we piloted the questionnaire, gathering feedback on content, wording, comprehension, and length. The language and wording of the survey were also assessed in relation to global appropriate meaning within different cultural contexts. The survey was initially developed in English before being translated into French, Greek, Spanish, Arabic, Brazilian Portuguese, Finnish, Urdu, Bengali, and Romanian.

The survey was available online from 2 June to 16 November 2020 to allow coverage of the early phase of the pandemic and (where relevant) lockdown across different nations. It was promoted through focused and purposeful email distribution, social media, traditional media, e-zines as well as through community, voluntary and charitable websites. Participants in the CLIC survey were required to be aged 18+ and able to provide informed consent and in order to participate, individuals must have been internet users. The exclusion criteria were non-consent, below 18, and could not complete the survey online. Participation in the study was voluntary and confidential, and no personal identifying information was collected.

### 2.1. Ethical Approval

The study was approved by the Ethics Committee of Ulster University (RG3) on 15 May 2020 and, where required, it was additionally ratified by ethics committees in participating countries.

### 2.2. Measures

#### 2.2.1. Loneliness: Pre-COVID-19 and during COVID-19 Pandemic

Loneliness was assessed by the modified 5-item UCLA loneliness scale, which has been validated in previous studies [27,28,29,30,31,32]. This included items relating to how often respondents feel “lonely” and “in tune with” the people around them (reverse scored) in addition to the 3 items in the short UCLA scale [33]. Response options were hardly ever (0), some of the time (1) and often (2), providing an overall score between 0 and 10 with higher scores denoting higher loneliness. We first asked the questions about “Before COVID-19” and later for “During COVID-19” giving us pre and during COVID-19 loneliness scores which were categorized as scores of 0–4 denoting none/low loneliness; 5–6 denoting moderate loneliness; and 7+ severe loneliness [34]. Cronbach’s alpha for the UCLA scale for the overall sample was 0.77 and 0.82 for pre and during COVID-19, respectively. Testing for scale reliability within WHO regions provided scores of 0.79 and 0.83, respectively, for UCLA pre and during COVID-19 in both Europe and Central Asia and North America.

#### 2.2.2. Social Isolation: Pre-COVID-19 and during COVID-19 Pandemic

Isolation prior to COVID-19 was captured using the six-item Lubben Social Network Scale (LSNS-6). Similar to the UCLA scale, the LSNS-6 has been validated internationally [35]. The LSNS-6 includes three items relating to family and three items relating friendships [35] with scores of 0 (none) to 5 (nine or more) for each item summed to provide an overall score between 0 and 30. Participants with scores <12 are defined as isolated. LSNS-6 items were preceded with “usually” to capture the respondent’s usual experience outside of COVID-19. Change in isolation before and after COVID-19 for each item was measured by a question if isolation was “about the same”, “more than usual” (+1), or “less than usual” (−1). Scores for these 6 items were summed to provide a score between −6 and 6, with scores of −3 or lower indicating a large increase in isolation during COVID-19. Cronbach’s alpha for the LSNS-6 was 0.82 in the sample overall, with a score of 0.75 for the scale assessing evaluations of change. Looking at scale reliability by WHO regions, scores of 0.82 and 0.81 were observed for the LSNS-6 in Europe and Central Asia and North America, respectively. In relation to evaluations of change, scores of 0.75 and 0.69 were observed in Europe and Central Asia and North America.

#### 2.2.3. Socio-Demographic and Other Characteristics

Demographic variables selected for analysis were gender (respondents were asked to self-classify their gender and a binary gender variable, with “other” (n = 37) and “prefer not to say” (n = 61) coded as missing was ultimately employed in models due to the low numbers in these categories); age (18–34; 35–44; 45–54; 55–69; 70+ years); education (none to elementary; secondary/diploma; degree +); how well needs were felt to be met by available financial resources (very well; fairly well; poorly); employment (employed/work from home; self-employed; education/training; unemployed/not working due to COVID-19; retired; and other/homemaker). To facilitate the global nature of the survey, a self-classification question establishing membership “of a minority group” (yes/no) was used, as was a subjective question of whether the respondent provides “care and support to a family member or friend with a long-term limiting or life-limiting health problem or disability” (yes/no). The importance of religion in participants’ lives was rated on a scale of 1 (not important at all) to 5 (very important). Additionally, we asked (a) if participants lived with others and if “living alone” was by choice; (b) how much time they spent alone pre-COVID-19 (always alone; often alone; seldom alone; never alone).

#### 2.2.4. Health

Physical and mental health were self-rated as excellent/very good/good/fair/poor with binary dummy variables employed in analysis, scoring responses of excellent, very good and good as 1 and fair or poor as 0. In relation to health behaviours (sleep; alcohol consumption; smoking and physical activity), respondents were asked to rate these during COVID-19 as “less than before”, “more than before” or “about the same” with response options of “do not partake” also available in relation to smoking and alcohol.

#### 2.2.5. Place and Community

Participants recorded country of residency, urban–rural location, and length of residency in the current area (1–4 years; 5–9 years; 10+ years). Additionally, we included a Likert-type item rating agreement with the statement that they lived in “a close-knit” or “tight” neighbourhood where people generally know one another on a scale from (1) strongly disagree to (5) strongly agree. 

We measured (1) frequency of speaking “to friends or family using online face-to-face technology” during and pre-COVID-19 (never/hardly ever; sometimes (less than weekly but at least monthly); often (at least weekly)); (2) Satisfaction with communication (“How satisfied are you with using online face to face technology as a form of communication” on a scale of 1 (very dissatisfied) to 5 (very satisfied).

#### 2.2.6. Experiences of Bereavement and/or COVID-19

Respondents reported (a) whether someone close to them had died during the pandemic due to any cause and (b) whether they or someone close had been diagnosed with COVID-19 (me; someone close; both; neither) or hospitalised (neither; me or someone close).

### 2.3. Analysis

Only surveys with completed consents were included in the analysis. Data were exported from Qualtrics and prepared and analysed in Stata 16.0 [36]. Prior to analysis, missing data were imputed for key outcome measures: UCLA pre-COVID-19 pandemic; UCLA during COVID-19 pandemic, LSNS-6 pre-COVID-19 pandemic; and scores of subjective evaluation of LSNS-6 change during the COVID-19 pandemic. For each of these outcomes *separately* missing values associated with their component variables were derived, with age, gender and the immediate associated components providing the basis for the derivations. This independence at the point of derivation ensured the derived values were generated in relation to only the components of that individual key outcome, ensuring no *contamination* across outcomes. This process used the Stata command *mi impute*. Relevant pre- and post-imputation distributions for each component variable showed them as broadly comparable [36].

Twenty-five exposure variables considered to be risk factors for either or both loneliness and/or social isolation were initially selected and grouped as above to create five models observing the potential impacts of (1) demographics; (2) health; (3) place; (4) communications; and connections (5) experiences of bereavement and/or COVID-19, all commonly cited factors in the literature [37,38,39,40]. The selected exposure variables combine macro (societal), meso (community/neighbourhood) and micro (individual) risk factors applicable within a global context and included both subjective and objective components. 

Our analytical strategy had three stages. First, models 1–5 were run for each of the four outcomes (UCLA pre-COVID-19 pandemic; UCLA during COVID-19 pandemic, LSNS-6 pre-COVID-19 pandemic; self-assessed change in isolation (LSNS-6) during COVID-19 pandemic). Multinomial logistic regression models (with none/low loneliness as the baseline comparison group) were used for UCLA models and logistic regression analysis for LSNS-6 outcomes to model the likelihood of being isolated pre COVID-19 or reporting a substantial increase in isolation during COVID-19.

As step two, we ran a final model for each outcome including predictors that were significant at the <0.05 level for models 1–5 during step one. Finally, in step three, all variables with a significant association at the <0.05 level in any of the models were included to identify common associations across final outcome variable models for UCLA scale pre-COVID-19 pandemic; UCLA scale during COVID-19 pandemic, LSNS-6 pre-COVID-19 pandemic; and self-assessed change of LSNS-6 during COVID-19 pandemic.

## 3. Results

At closure of the survey, 23,609 respondents from 101 different countries had participated; of these, 20,398 responses were assessed as valid (i.e., completion of written informed consent). The number of respondents in the analytic samples in logistic regression models ranged from 9618 to 10,485, due to missing data. Just over a third (39%) of respondents (n = 14,302) completed all items in the survey. (This level of partial response observed is common in online surveys [41].)

The sample was predominantly female (79%), highly educated (75% educated to degree level) and composed of older adults (Mean = 53 years (SD 17.6) Minimum: age 18, Maximum age: 104). Forty percent of the sample were resident in the United States, 21% in the UK and Ireland, 6% in Pakistan and 5% in Mexico. Samples from the remaining countries were less than 5% of the total sample. Caregivers made up 27.5% of the sample; 11% described themselves as belonging to a “minority group”; most were urban dwellers (52%) and 82% reported being in excellent or good mental health (Table 1).

The prevalence of severe loneliness was 21% during COVID-19 compared with 6% prior to COVID-19. A fifth (21%) were defined as isolated based on their usual connections, with 13% reporting a substantial increase in isolation based on participants’ evaluation of change during COVID-19. Socio-demographic and other characteristics, outcomes and prevalence of all exposure variables of the respondent sample are provided in Table 1 and CLIC responses by WHO Regions are provided in Table A1 in the Appendix A.

(1) Loneliness:

For both pre and during COVID-19 loneliness, those whose financial resources did not meet their needs; with poor physical or mental health; who did not rate their neighbourhood as close knit; living in their neighbourhood for less than 4 years; and living alone not by choice demonstrated increased odds of severe/moderate loneliness. The following factors increased the risk of experiencing loneliness during COVID-19: being female, unemployed/in education or training, increased alcohol consumption, decreased physical activity, worse sleep and dissatisfaction with video calls (see Table 2 and Table 3).

(2) Social isolation:

Factors associated with social isolation, both pre and during COVID-19, were those whose financial resources did not met their needs, had poor self-rated physical and mental health and who indicated the personal importance of religion. Specific factors during COVID-19 associated with increased isolation were worse sleep, less physical activity, alcohol consumption, dissatisfaction with videocalls, living alone not by choice, time alone, homemakers and carers (see Table 4 and Table 5).

The final regression models for each of the 4 outcomes are presented in Table 2, Table 3, Table 4 and Table 5: pre-COVID-19 loneliness (UCLA); during COVID-19 loneliness (UCLA); pre-COVID-19 isolation (LSNS-6) and perceived substantial increase in isolation during COVID-19 (LSNS-6).

## 4. Discussion

To our knowledge, this survey on loneliness and social isolation during the pandemic is the largest and most international of its kind. Our transnational data permitted an examination of loneliness and social isolation (separately and comparatively) both before and during COVID-19 at a global level, with pre and during COVID-19 evaluations of loneliness and isolation measured using established scales. 

The association between loneliness and living alone is well established in the literature [42,43,44,45], with a significant rise in the percentage of people living alone across most Western capitalist countries [46]. A key finding of the current study is that there was more than a three-fold increase in participants’ reporting of severe loneliness from a perceived pre COVID-19 state. Moreover, severe loneliness is hugely elevated among those who describe it as involuntary. Thus, during the pandemic, living alone was associated with 61% higher loneliness among those who lived alone by choice but nearly seven times higher among those living alone not by choice. 

Younger age, low educational attainment, poor physical and mental health, financial insecurity, perceived low community support and lower importance of religion are all independently and significantly associated with risk of loneliness. We discuss these and other factors in turn. Those who reported poorer mental health also showed greater risk, consistent with other evidence on loneliness and mental health in general [47] as well as risk of loneliness during the pandemic specifically [15,18,47]; indeed, we noted a four-fold increased risk of severe loneliness during the pandemic. As others have suggested, the relationships and pathways between poor mental health and loneliness are often connected, with many shared and socially excluding factors linked to both conditions, e.g., stigma, anxiety, low self-esteem and motivation [30]. As with other studies [15,21], we found that people most likely to report loneliness are those who also considered themselves financially insecure. Interestingly, the effect of financial insecurity appeared to be reduced when considered during COVID-19 but remained significant, suggesting perhaps that the impact of the pandemic had a perceived equalizing effect on people’s perceptions of difference. In the U.S., for example, there were increases in social safety net funding during the pandemic, in large part to protect the economy. Temporary policies regarding rent and loan payment deferrals were also introduced. Similar financial “cushions” were introduced in the UK. It is not clear to what extent such policies assisted with their desired outcome. 

While much of the literature on loneliness and isolation is based on research with older people [37,43,44,48], we found that pre COVID-19, older people tended to be significantly less prone to loneliness, with the greatest reduction in risk noted among people aged 70 years and above, which may be due to a lowering of social needs and/or expectations as one ages, or a cohort of relatively more resilient older adults. Additionally, while homemakers and those in education/training reported loneliness, before COVID-19, only unemployment and being retired appeared to increase the risk of loneliness during the pandemic, perhaps suggesting a relationship with the loss of social relationships provided by work. In line with other studies, carers were at increased risk of loneliness and an increase in social isolation during COVID-19. This would indicate that there was a deficit in social connections during the pandemic, which was not filled by the connection of being a caregiver. Interestingly too, the direct impact of COVID-19 through hospitalisation of either self or others appeared to have little impact on isolation [49].

Previous research concluded that low social capital, especially in terms of low trust, may be a risk factor for loneliness [50]. In the CLIC survey, social capital also appears as a consistently strong factor, whereby people’s perceptions of their neighbourhood as “tight or close-knit” is inversely related to feelings of loneliness before and during the pandemic and social isolation before the pandemic. We noted with interest that social isolation as measured by the LSNS-6 Scale [35] shows much stronger effects for locale and neighbourhood than those detected through the UCLA. Thus, we noted a strong urban–rural divide with rural/town-dwellers significantly more at risk compared to city-dwellers during the pandemic. This seems to contradict other evidence on an urban/rural divide on loneliness [51,52,53] but may in fact highlight their differences as separate constructs—thus, people in rural areas may accept their geographical isolation but are not necessarily lonely. As with loneliness, those who strongly disagreed that they live in a close-knit community had a three-fold risk of feeling isolated pre COVID-19.

The importance of satisfaction with video connection became significantly heightened during COVID-19. These data showed that greater dissatisfaction with video calls during the pandemic was associated with significantly higher loneliness and social isolation (compared to those who were very satisfied). Again, this study demonstrates the complexity of digital communication technology to attenuate people’s sense of isolation and loneliness. However, unfortunately, the survey did not include a not applicable category for those who do not use (or do not want to use) video calls because they find it an unsatisfactory form of communication or do not have the skills or technology to use video calls.

Religious involvement is thought to be protective for mental health [54], with previous studies on the relationship between loneliness, mental health and religion [55,56] pointing to the social dimensions of religion (e.g., attendance). We found that the greater the personal significance of religion, the likelihood of loneliness and isolation (at all levels) increased significantly, except for participants’ assessment of pre-COVID isolation, whereby we noted that religion was significantly protective. These findings suggest that the closure of places of worship during the pandemic impacted considerably on social connectedness [57]. It may also indicate that the pandemic provoked a much greater reflection on death and spirituality [58].

Reporting on changes to health behaviours during COVID-19, we found that sleep is strongly associated with loneliness, whereas alcohol consumption has no clear direction and may be connected to both reduced and increased intake. However, our findings suggest that loneliness is more strongly associated with increased use of alcohol. Current US evidence suggests that during the summer of 2020, adults who felt lonelier on average across 30 days consumed more alcohol each day. However, on days when they felt lonelier than usual, they reported drinking less alcohol [59].

The overarching finding from the CLIC data is that personal finances and mental health are a consistently cross-cutting aspect of loneliness and social isolation both before and during the pandemic, impacting the most vulnerable groups and reinforcing existing inequalities. This is consistent with emerging evidence that indicates a widening of pre-existing inequalities during COVID-19 [15].

### Strengths and Limitations

This large transnational survey has over 20,000 responses aged 18+ from 101 countries and 10 languages, and this particular paper has an analytic sample of 9618–10,485 respondents from at least 70 countries. Each survey was translated by Qualtrics and checked by a country lead. It uses scales that have previously been validated in a majority of the languages and cultures represented in the CLIC study. Individual country and language psychometric analyses of the major scales are currently underway and papers on cross-cultural and national differences are being planned. This study has allowed us to examine and understand personal and social risk factors for loneliness both before and during the COVID-19 pandemic, taking into account a range of contextual and risk factors for social isolation pre-pandemic, as well as those associated with a large increase in social isolation during COVID-19. However, despite the size and breadth of the data, our findings are based on a non-representative sample of people who completed the online questionnaire. This method is, of course, prone to bias. For example, people who felt particularly lonely may be drawn to the survey. Additionally, completion online excludes people without access to the internet for various reasons (e.g., ICT literacy, living in remote and rural areas or financial disadvantage), each of which may be implicitly biased. This may mean that the results understate the impact of the pandemic on the most vulnerable in society as ICT usage reflects existing inequalities [60]. Additionally, “pre-COVID-19” responses are retrospective and may be prone to recall error. Moreover, we were unable to explore health and health behaviours in detail. Our sample was predominantly female, highly educated and older and despite the breadth of the transnational sample, some national groups have greater representation. Lastly, we were unable to compare the various countries’ public health and social measures introduced during COVID-19 and their impact on loneliness and social isolation.

## 5. Conclusions

The COVID-19 pandemic has had a global impact—however, it has not impacted everyone equally. Government responses of “social distancing” have presented massive challenges in maintaining connections and managing feelings of loneliness and experiences of social isolation for particularly vulnerable groups—those with inadequate personal finances and poor mental health. 

Addressing inequalities is a key objective of public health interventions but unfortunately, it is not readily accommodated within many of the interventions to address loneliness and social isolation. If we wish to benefit the greatest number of people, there is a need for a major rethink in policy and service provision. Greater emphasis is required on primary prevention and on population-based strategies to promote social health and recognize the impact of physical and mental well health, living conditions and socioeconomic factors [1,61]. Our study offers new insights into understanding the experience of loneliness and social isolation both before and during the pandemic and provides the basis to help develop a more sophisticated understanding to better guide policy in this area. We show the core issues that need to be addressed. Public health approaches are needed to address the root causes of loneliness and social isolation and support adequate income, social engagement and connections, healthy behaviours and address the needs of specific groups such as carers or those living alone. While many current interventions show little evidence of effectiveness [62], we know that many of the factors associated with loneliness and isolation are mutable and should be central to social equality and justice policies, which can influence community solidarity and individual resilience. 

## Figures and Tables

**Table 1:** Profile of CLIC sample (n = 23,609).

Variable (N Completed Item)	N (%)
**Gender (n = 18,991)** Females	14,917 (79%)
**Age** (n = 17,436)	Mean = 53 (SD 17.6)
**Education** (n = 18,056) **Degree** +	13,504 (75%)
**Needs met by financial resources** (n = 18,231) Very well Fairly well Poorly	8046 (44%) 8456 (46%) 1729 (10%)
**Member of minority group** (n = 18,353) Yes	1977 (11%)
**Care provider** (n = 19,046) Yes	5236 (27.5%)
**Employment status** (n = 18,295) Employed including work from home Self employed In education/training Unemployed Retired Other/homemaker	7324 (40%) 1084 (6%) 1233 (7%) 1419 (8%) 6039 (33%) 1196 (6.5%)
**Self-rated physical health** (n = 18,236) Excellent/Very good/good Fair/poor	15,728 (86%) 2508 (14%)
**Self-rated mental health** (n = 18,212) Excellent/Very good/good Fair/poor	14,873 (82%) 3339 (18%)
**Quality of sleep during COVID-19** (n = 17,852) Worse than before About the same	5905 (33%) 10,668 (60%)
**Physical activity during COVID-19** (n = 17,840) Less than before About the same	8677 (49%) 5909 (33%)
**Smoking during COVID-19** (n = 17,852) Less than before More than before About the same	271 (1.5%) 709 (4%) 708 (4%)
**Urban/rural** (n = 18,277) City Town Rural	9512 (52%) 5076 (28%) 3689 (20%)
**Lived in current area** (n = 18,272) 1–4 years 5–9 years 10+ years	2940 (16%) 2125 (12%) 13,207 (72%)
**Close-knit/“tight” neighbourhood** (n = 15,922) Strongly disagree	1144 (7%)
**Use of face-to-face technology pre-COVID-19** (n = 17,055) Never/Hardly ever	9213 (54%)
**Use of face-to-face technology during COVID-19** (n = 16,973) Never/Hardly ever	5490 (32%)
**Satisfied with face-to-face technology for communication** (n = 15,016) Very dissatisfied	1116 (7%)
**Living with others** (n = 17,231) Yes	13,374 (78%)
**Time spent alone pre COVID-19** (n = 17,134) Always and often alone Seldom and never alone	6748 (39%) 10,386 (61%)
**Importance of religion** (n = 14,050) Not important at all Very important	4756 (34%) 4049 (29%)
**Someone close died at this time** (n = 18,122) Yes	3274 (18%)
**Me or someone close diagnosed with COVID-19** (n = 18,120)	3359 (18.5%)
**Me or Someone close Hospitalised with COVID-19** (n = 18,097) Me/ someone close	1562 (9%)
Outcomes	N (%)
**UCLA pre-COVID pandemic** (n = 16,452) None/low (0–4) Moderate (5–6) Severe (7+)	13,204 (80%) 2314 (14%) 934 (6%)
**UCLA during COVID pandemic** (n = 16,343) None/low (0–4) Moderate (5–6) Severe (7+)	9277 (57%) 3659 (22%) 3407 (21%)
**Lubben pre-COVID pandemic** (n = 15,408) Isolated (<12) Not isolated	3188 (21%) 12,220 (79%)
**Lubben change during COVID pandemic** (n = 15,322) Large increase in isolation (score < −2) Small or no increase in isolation	1989 (13%) 13,333 (87%)

**Table 2:** Final multinomial logistic regression model for moderate and severe loneliness pre COVID-19 (UCLA).

Base: None/Low Loneliness (UCLA = 0–4)	Moderate Loneliness (UCLA = 5–6)	Severe Loneliness (UCLA 7+)
	RRR 95% CI	RRR 95% CI * Significance
18–34 (ref) 35–44 45–54 55–69 70 and over	1.00 1.02 (0.78–1.32) 0.80 (0.62–1.03) 0.72 (0.57–0.91) ** 0.58 (0.42–0.78) ***	1.00 1.07 (0.72–1.59) 0.63 (0.42–0.94) * 0.66 (0.46–0.95) * 0.48 (0.30–0.77) **
**Female**	1.81 (0.99–1.40)	1.10 (0.84–1.43)
**Education** (ref: Degree or higher) Secondary/diploma To elementary	1.00 1.02 (0.87–1.19) 1.21 (0.66–2.24)	1.00 1.20 (0.96–1.51) 0.54 (0.19–1.51)
**Finances meet needs**: (ref: very well) Fairly well Poorly	1.00 1.30 (1.12–1.50) *** 1.55 (1.22–1.97) ***	1.00 1.57 (1.23–2.00) *** 3.31 (2.40–4.58) ***
**Member of minority**	1.06 (0.85–1.30)	0.87 (0.62–1.21)
**Employment status** (ref: employed including work from home) Self employed In education/training Unemployed/not working Retired Other/homemaker	1.00 0.86 (0.65–1.18) 1.30 (0.97–1.74) 1.08 (0.84–1.40) 0.96 (0.77–1.18) 1.08 (0.83–1.42)	1.00 0.93 (0.57–1.50) 1.73 (1.14–2.63) * 1.24 (0.86–1.81) 1.21 (0.86–1.70) 1.68 (1.16–2.44) **
**Self-rated mental health** Fair/poor	2.61 (2.23–3.07) ***	5.27 (4.19–6.63) ***
**Self-rated physical health** Fair/poor	1.49 (1.24–1.79) ***	1.32 (1.02–1.71) *
**Sleep during COVID-19** (ref: about the same) Better than before Worse than before	1.00 1.32 (1.02–1.72) * 1.35 (1.17–1.56) ***	1.00 1.50 (1.00–2.25) 1.08 (0.86–1.35)
**Physical activity COVID-19** (ref: about the same) Less than before More than before	1.00 1.17 (1.00–1.36) 1.09 (0.89–1.34)	1.00 0.96 (0.76–1.22) 0.99 (0.72–1.37)
**Alcohol consumption during COVID-19** (ref: about the same) Less than before More than before Do not partake	1.00 0.84 (0.67–1.07) 0.99 (0.81–1.22) 1.03 (0.87–1.21)	1.00 0.94 (0.66–1.35) 0.99 (0.71–1.38) 1.27 (0.99–1.64)
**Urban** (ref: city) Town Rural	1.00 0.99 (0.85–1.17) 1.13 (0.95–1.35)	1.00 1.14 (0.89–1.44) 1.10 (0.83–1.44)
**Close-knit neighbourhood** (ref: 5 (Strongly agree)) 1 (Strongly disagree) 2 3 4	1.00 2.05 (1.52−2.75) *** 1.61 (1.26−2.07) *** 1.66 (1.31−2.10) *** 1.14 (0.88−1.48)	1.00 1.62 (1.06−2.48) * 1.33 (0.92−1.92) 0.98 (0.69−1.41) 0.94 (0.64−1.38)
**Time in area** (ref: 10 years+) 1–4 years 5–9 years	1.00 1.29 (1.08−1.55) ** 1.11 (0.90−1.36)	1.00 1.49 (1.15−1.93) ** 0.99 (0.71−1.39)
**Pre COVID-19 video calls** (ref: often) Hardly ever/never Sometimes	1.00 0.68 (0.54−0.85) ** 0.88 (0.70−1.09)	1.00 1.01 (0.71−1.46) 0.78 (0.53−1.13)
**During COVID-19 video calls** (ref: often) Hardly ever/never Sometimes	1.00 1.79 (1.44−2.23) *** 1.36 (1.13−1.63) **	1.00 1.55 (1.11−2.16) * 1.02 (0.75−1.38)
**Satisfaction with video calls** (ref: 5 (very satisfied)) 1 (very dissatisfied) 2 3 4	1.00 1.51 (1.13−2.01) * 1.31 1.04−1.67) * 1.16 (0.93−1.44) 1.13 (0.94−1.35)	1.00 1.44 (0.95−2.17) 1.44 (1.01−2.06) * 1.21 (0.86−1.71) 1.05 (0.78−1.41)
**Living alone** (ref: lives with others) Lives alone by choice Lives alone not by choice	1.00 0.85 (0.71−1.02) 1.57 (1.18−2.09) **	1.00 0.66 (0.49−0.87) ** 2.70 (1.91−3.81) ***
**Pre-COVID-19 time alone** (ref: always) Often Seldom Never	1.00 0.88 (0.58−1.35) 0.38 (0.24−0.58) *** 0.29 (0.18−0.48) ***	1.00 0.30 (0.19−0.45) *** 0.06 (0.04−0.10) *** 0.11 (0.06−0.19) ***
**Importance of religion** (ref: 1 (not at all important)) 2 3 4 5 (very important)	1.00 1.07 (0.86−1.32) 1.01 (0.79−1.28) 1.26 (1.03−1.54) * 1.17 (0.98−1.40)	1.00 0.99 (0.71−1.36) 1.08 (0.76−1.53) 0.92 (0.67−1.27) 0.83 (0.63−1.11)

*** = *p* < 0.001; ** = *p* < 0.005; * = *p* < 0.05.

**Table 3:** Final multinomial logistic regression model for moderate and severe loneliness during COVID-19 (UCLA).

Base: None/Low Loneliness (UCLA = 0–4)	Moderate Loneliness (UCLA = 5–6)	Severe Loneliness (UCLA 7+)
	RRR 95% CI	RRR 95% CI * Significance
18−34 (ref) 35−44 45−54 55−69 70 and over	1.00 0.84 (0.68−1.05) 0.72 (0.58−0.88) ** 0.84 (0.69−1.02) 0.81 (0.64−1.03)	1.00 0.99 (0.77−1.28) 0.77 (0.60−0.98) * 1.01 (0.80−1.26) 0.99 (0.75−1.30)
**Female**	1.26 (1.10−1.43) **	1.67 (1.42−1.97) ***
**Education** (ref: Degree or higher) Secondary/diploma To elementary	1.00 1.23 (1.09−1.39) ** 1.51 (0.93−2.45)	1.00 0.91 (0.79−1.06) 0.56 (0.29−1.09)
**Finances meet needs**: (ref: very well) Fairly well Poorly	1.00 1.31 (1.17−1.46) *** 1.16 (094−1.44)	1.00 1.42 (1.25−1.62) *** 1.61 (1.29−2.02) ***
**Provides Care**	1.42 (1.26−1.61) ***	1.46 (1.27−1.68) ***
**Employment status** (ref: employed including work from home) Self employed In education/training Unemployed/not working Retired Other/homemaker	1.00 0.97 (0.77−1.21) 1.17 (0.91−1.50) 1.53 (1.24−1.90) *** 1.07 (0.91−1.26) 1.04 (0.83−1.32)	1.00 0.77 (0.58−1.02) 1.24 (0.92−1.67) 1.80 (1.42−2.28) *** 1.35 (1.12−1.63) ** 1.28 (0.99−1.64)
**Self−rated mental health** (Fair/poor)	1.93 (1.65−2.27) ***	4.48 (3.83−5.24) ***
**Self−rated physical health** (Fair/poor)	1.05 (0.89−1.25)	1.24 (1.04−1.48) *
**Sleep during COVID-19** (ref: about the same) Better than before Worse than before	1.00 0.95 (0.77−1.17) 1.85 (1.65−2.08) **	1.00 0.94 (0.72−1.22) 2.47 (2.17−2.81) ***
**Physical activity during COVID-19** (ref: about the same) Less than before More than before	1.00 1.39 (1.23−1.56) *** 1.02 (0.88−1.19)	1.00 2.01 (1.75−2.32) *** 1.16 (0.96−1.40)
**Smoking during COVID-19** (ref: about the same) Less than before More than before Do not partake	1.00 0.68 (0.40−1.17) 0.98 (0.65−1.47) 0.98 (0.74−1.30)	1.00 0.91 (0.51−1.64) 1.56 (1.00−2.43) 1.08 (0.76−1.51)
**Alcohol consumption during COVID-19** (ref: about the same) Less than before More than before Do not partake	1.00 1.14 (0.95−1.37) 1.34 (1.14−1.58) *** 1.07 (0.94−1.22)	1.00 1.36 (1.07−1.66) ** 1.85 (1.55−2.20) *** 1.15 (0.99−1.33)
**Close−knit neighbourhood** (ref: 5 (Strongly agree)) 1 (Strongly disagree) 2 3 4	1.00 1.62 (1.28−2.05) *** 1.31 (1.09−1.58) ** 1.38 (1.16−1.64) *** 1.06 (0.88−1.27)	1.00 1.55 (1.19−2.00) ** 1.18 (0.96−1.44) 1.05 (0.87−1.28) 0.91 (0.74−1.11)
**Time in area** (ref: 10 years+) 1−4 years 5−9 years	1.00 1.17 (1.01−1.36) * 1.07 (0.91−1.26)	1.00 1.55 (1.32−1.82) *** 1.07 (0.88−1.28)
**Pre COVID-19 video calls** (ref: often) Hardly ever/never Sometimes	1.00 0.89 (0.77−1.03) 1.02 (0.88−1.19)	1.00 1.03 (0.87−1.23) 1.02 (0.85−1.23)
**Satisfaction with video calls** (ref: 5 (very satisfied)) 1 (very dissatisfied) 2 3 4	1.00 1.57 (1.23−1.99) *** 1.99 (1.65−2.40) *** 1.56 (1.32−1.84) *** 1.44 (1.26−1.65) ***	1.00 2.31 (1.80−2.96) *** 2.54 (2.07−3.11) *** 1.63 (1.35−1.97) *** 1.30 (1.11−1.52) **
**Living alone and choice** (ref: lives with others) Lives alone by choice Lives alone not by choice	1.00 1.31 (1.12−1.52) ** 2.36 (1.75−3.18) ***	1.00 1.61 (1.37−1.90) *** 6.82 (5.19−8.95) ***
**Pre COVID-19 time alone** (ref: always) Often Seldom Never	1.00 1.51 (1.01−2.26) * 1.25 (0.83−1.87) 1.19 (0.77−1.83)	1.00 1.13 (0.77−1.67) 0.97 (0.66−1.44) 0.68 (0.44−1.07)
**Importance of religion** (ref: 1 (not at all important)) 2 3 4 5 (very important)	1.00 1.28 (1.08−1.52) ** 1.37 (1.13−1.66) ** 1.27 (1.07−1.49) * 1.45 (1.26−1.68) ***	1.00 1.55 (1.28−1.86) *** 1.37 (1.10−1.70) * 1.39 (1.15−1.67) ** 1.53 (1.30−1.80) ***
**Hospitalised with COVID-19** (ref: neither) Respondent or someone close to respondent	1.00 1.21 (0.97−1.52)	1.00 0.99 (0.76−1.28)
**Diagnosed with COVID-19** (ref: neither) Respondent Someone close to respondent Both	1.00 0.67 (0.41−1.08) 0.90 (0.76−1.06) 1.31 (0.82−2.10)	1.00 0.63 (0.37−1.08) 1.00 (0.83−1.20) 1.18 (0.67−2.07)
**Someone close to respondent died at this time due to any cause**	1.02 (0.88−1.17)	1.11 (0.95−1.30)

*** = *p* < 0.001; ** = *p* < 0.005; * = *p* < 0.05.

**Table 4:** Final logistic regression model for isolation pre COVID-19 (LSNS-6).

	OR 95% CI * Significance
18–34 35−44 45−54 55−69 70 and over	1.00 1.06 (0.84−1.34) 1.04 (0.83−1.30) 1.20 (0.98−1.47) 0.97 (0.75−1.25)
**Female**	0.72 (0.63−0.82) ***
**Education** (ref: Degree or higher) Secondary/diploma To elementary	1.00 1.17 (1.03−1.33) * 2.99 (1.85−4.82) ***
**Finances meet needs** (ref: very well) Fairly well Poorly	1.00 1.28 (1.13−1.44) *** 1.42 (1.15−1.74) **
**Member of minority**	1.12 (0.94−1.34)
**Employment status** (ref: employed including work from home) Self employed In education/training Unemployed/not working Retired Other/homemaker	1.00 0.89 (0.69−1.15) 1.29 (0.99−1.67) 1.10 (0.88−1.37) 0.95 (0.79−1.13) 1.01 (0.80−1.27)
**Self−rated mental health** (Fair/poor)	2.06 (1.78−2.37) ***
**Self−rated physical health** (Fair/poor)	1.28 (1.09−1.50) **
**Physical activity during COVID-19** (ref: about the same) Less than before More than before	1.00 1.20 (1.06−1.36) * 1.03 (0.87−1.23)
**Alcohol consumption during COVID-19** (ref: about the same) Less than before More than before Do not partake	1.00 0.77 (0.62−.95) * 0.99 (0.82−1.18) 1.43 (1.24−1.63) ***
**Urban/rural** (ref: city) Town Rural	1.00 1.27 (1.11−1.45) *** 1.28 (1.11−1.49) **
**Close−knit neighbourhood** (ref: 5 (Strongly agree)) 1 (Strongly disagree) 2 3 4	1.00 3.01 (2.37−3.81) *** 1.82 (1.49−2.23) *** 1.47 (1.21−1.79) *** 0.99 (0.80−1.22)
**During COVID video calls** (ref: often) Hardly ever/never Sometimes	1.00 2.49 (2.12−2.91) *** 1.26 (1.09−1.47) **
**Satisfaction with video calls** (ref: 5 (very satisfied)) 1 (very dissatisfied) 2 3 4	1.00 1.49 (1.19−1.88) ** 1.24 (1.01−1.51) * 1.12 (0.94−1.35) 1.06 (0.91−1.23)
**Living alone and choice** (ref: lives with others) Lives alone by choice Lives alone not by choice	1.00 1.01 (0.87−1.18) 1.15 (0.90−1.48)
**Pre−COVID time alone** (ref: always) Often Seldom Never	1.00 0.45 (0.33−0.62) *** 0.26 (0.19−0.36) *** 0.61 (0.43−0.88) *
**Importance of religion** (ref: 1 (not at all important)) 2 3 4 5 (very important)	1.00 0.78 (0.65−0.94) * 0.76 (0.62−0.94) * 0.74 (0.92−0.88) ** 0.76 (0.65−0.88) ***
**Hospitalised with COVID-19** (ref: neither) Respondent or someone close to respondent	1.00 1.21 (0.94−1.55)
**Diagnosed with COVID-19** (ref: neither) Respondent Someone close to respondent Both	1.00 0.70 (0.42−1.18) 0.76 (0.63−0.91) ** 1.67 (1.06−2.62) *
**Someone close to respondent died at this time due to any cause**	0.92 (0.79−1.08)

*** = *p* < 0.001; ** = *p* < 0.005; * = *p* < 0.05.

**Table 5:** Final logistic regression model for perceived large increase in isolation during COVID-19.

	OR 95% CI * Significance
18–34 35−44 45−54 55−69 70 and over	1.00 1.06 (0.84−1.32) 0.96 (0.78−1.20) 0.79 (0.64−0.97) 0.59 (0.45−0.79) ***
**Female**	1.10 (0.94−1.29)
**Finances meet needs** (ref: Very well) Fairly well Poorly	1.00 1.25 (1.10−1.43) ** 1.57 (1.27−1.93) ***
Provides care	1.29 (1.13−1.48) ***
**Employment status** (ref: employed including work from home) Self employed In education/training Unemployed/not working Retired Other/homemaker	1.00 0.91 (0.69−1.19) 1.40 (1.09−1.80) * 1.09 (0.87−1.37) 0.90 (0.74−1.10) 1.44 (1.15−1.80) **
**Self−rated mental health** (Fair/poor)	1.25 (1.07−1.47) *
**Self−rated physical health** (Fair/poor)	1.28 (1.08−1.52) *
**Sleep during COVID-19** (ref: about the same) Better than before Worse than before	1.00 1.08 (0.85−1.38) 1.55 (1.36−1.77) ***
**Physical activity during COVID-19** (ref: about the same) Less than before More than before	1.00 1.38 (1.20−1.59) *** 0.95 (0.79−1.15)
**Alcohol consumption during COVID-19** (ref: about the same) Less than before More than before Do not partake	1.00 1.45 (1.18−1.78) *** 1.37 (1.14−1.65) ** 1.23 (1.05−1.43) *
**Pre COVID-19 video calls** (ref: often) Hardly ever/never Sometimes	1.00 0.93 (0.79−1.10) 0.98 (0.82−1.17)
**Satisfaction with video calls** (ref: 5 (very satisfied)) 1 (very dissatisfied) 2 3 4	1.00 1.76 (1.35−2.28) *** 2.07 (1.69−2.54) *** 1.40 (1.15−1.71) ** 1.54 (1.31−1.81) ***
**Living alone and choice** (ref: lives with others) Lives alone by choice Lives alone not by choice	1.00 0.90 (0.74−1.08) 1.68 (1.29−2.20) ***
**Pre−COVID time alone** (ref: always) Often Seldom Never	1.00 1.56 (0.97−2.51) 2.08 (1.29−3.36) * 1.84 (1.10−3.06) *
**Importance of religion** (ref: 1 (not at all important)) 2 3 4 5 (very important)	1.00 1.40 (1.15−1.70) ** 1.32 (1.06−1.65) * 1.37 (1.13−1.66) ** 1.62 (1.37−1.91) ***
**Hospitalised with COVID-19** (ref: neither) Respondent or someone close to respondent	1.00 0.89 (0.72−1.11)
**Someone close to respondent died at this time due to any cause**	1.03 (0.88−1.21)

*** = *p* < 0.001; ** = *p* < 0.005; * = *p* < 0.05.

## Data Availability

Restrictions apply to the availability of the CLIC data. To request data access, readers should contact the lead author R.O.

## Appendix A

**Table A1:** CLIC Sample by WHO Regions (n = 17,701).

	N (%)
East Asia and Pacific	456 (2.6%)
Europe and Central Asia	5739 (32%)
Latin America and the Caribbean	1580 (9%)
Middle East and North Africa	207 (1%)
North America	7770 (44%)
South Asia	1861 (10.5%)
Sub-Saharan Africa	88 (0.50%)
